# Long-term nutrient inputs shift soil microbial functional profiles of phosphorus cycling in diverse agroecosystems

**DOI:** 10.1038/s41396-019-0567-9

**Published:** 2019-12-11

**Authors:** Zhongmin Dai, Guofei Liu, Huaihai Chen, Chengrong Chen, Jingkuan Wang, Shaoying Ai, Dan Wei, Daming Li, Bin Ma, Caixian Tang, Philip C. Brookes, Jianming Xu

**Affiliations:** 1 Institute of Soil and Water Resources and Environmental Science, College of Environmental and Resource Sciences, Zhejiang University, Hangzhou, 310058 China; 2 Zhejiang Provincial Key Laboratory of Agricultural Resources and Environment, Zhejiang University, Hangzhou, 310058 China; 3 The Rural Development Academy, Zhejiang University, Hangzhou, 310058 China; 4 Biological Sciences Division, Pacific Northwest National Laboratory, Richland, WA 99354 USA; 5 Australian Rivers Institute, School of Environment and Sciences, Griffith University, Nathan Campus, Brisbane, QLD 4111 Australia; 6 College of Land and Environment, Shenyang Agricultural University, No. 120 Dongling Road, Shenhe District, Shenyang, 110866 Liaoning China; 7 Institute of Agricultural Resources and Environment, Guangdong Academy of Agricultural Sciences, Guangzhou, 510640 Guangdong China; 8 Soil Fertilizer and Environment Resource, Heilongjiang Academy of Agricultural Sciences, Haerbin, 150086 Heilongjiang China; 9 Jiangxi Institue of Red Soil, Jiangxi Key Laboratory of Red Soil Arable Land Conservation, Jinxian, 331717 China; 10 Department of Animal, Plant and Soil Sciences, Centre for AgriBioscience, La Trobe University, Bundoora, VIC 3086 Australia

**Keywords:** Metagenomics, Microbial ecology, Biogeochemistry, Functional genomics

## Abstract

Microorganisms play an important role in soil phosphorus (P) cycling and regulation of P availability in agroecosystems. However, the responses of the functional and ecological traits of P-transformation microorganisms to long-term nutrient inputs are largely unknown. This study used metagenomics to investigate changes in the relative abundance of microbial P-transformation genes at four long-term experimental sites that received various inputs of N and P nutrients (up to 39 years). Long-term P input increased microbial P immobilization by decreasing the relative abundance of the P-starvation response gene (*phoR*) and increasing that of the low-affinity inorganic phosphate transporter gene (*pit*). This contrasts with previous findings that low-P conditions facilitate P immobilization in culturable microorganisms in short-term studies. In comparison, long-term nitrogen (N) input significantly decreased soil pH, and consequently decreased the relative abundances of total microbial P-solubilizing genes and the abundances of *Actinobacteria*, *Gammaproteobacteria*, and *Alphaproteobacteria* containing genes coding for alkaline phosphatase, and weakened the connection of relevant key genes. This challenges the concept that microbial P-solubilization capacity is mainly regulated by N:P stoichiometry. It is concluded that long-term N inputs decreased microbial P-solubilizing and mineralizing capacity while P inputs favored microbial immobilization via altering the microbial functional profiles, providing a novel insight into the regulation of P cycling in sustainable agroecosystems from a microbial perspective.

## Introduction

Phosphorus (P) is an essential macronutrient for all biota, while a large proportion of P is immobilized to relatively unavailable inorganic and organic forms in agricultural soils [1]. Therefore, a small amount of remaining available P cannot meet the needs of microbial or plant growth [2, 3]. Microorganisms play a crucial role in soil P cycling and in regulating P availability [4]. The microbial P-transformation processes are mainly mediated by three microbial gene groups, namely, genes involved in inorganic P-solubilization and organic P-mineralization, P-uptake and transport, and P-starvation response regulation [5].

The microorganisms containing genes involved in inorganic P-solubilization and organic P-mineralization can release organic anions to solubilize inorganic P or enzymes to mineralize organic P. A typical gene involved in solubilizing inorganic P is the gene (*gcd*) coding for quinoprotein glucose dehydrogenase (PQQGDH). It directly governs the oxidation pathway of glucose and acidification of the periplasmic space [6]. In addition, microorganisms containing genes coding for enzymes, such as alkaline phosphatase (*phoD* and *phoA*), phytase (*appA*), and C-P lyases (*phn*), have high capacities to mineralize organic-P compounds in soils [7]. In contrast, microorganisms containing genes coding for P-uptake and transport systems, i.e., high-affinity (*pst)* and low-affinity *(pit)* transporters, can assimilate inorganic P under the P-low and P-rich conditions, respectively [8]. These genes enable microorganisms to efficiently utilize P and to immobilize P into their biomass, and may compete for the available P with plants in agroecosystems [9]. The genes involved in P-starvation response regulation (*phoU*, *phoR*, and *phoB*) enable microorganisms to utilize external P sources. These genes connect strongly with the genes involved in P-uptake and transport (e.g., *pst)* and control the expression of genes coding for alkaline phosphatase [10], especially under low-P condition [8].

Nitrogen (N) and P inputs are considered as an important measure to maintain soil fertility and crop yields [11]. The inputs of these nutrients are increasing rapidly in agroecosystems across the globe, which alters soil N:P stoichiometry and other properties such as pH [12, 13], leading to the changes in size, structure, diversity, and activity of the soil microbial community [14–17] and microbial functional traits responsible for C and N cycling [18, 19]. However, few studies have investigated the effects of long-term P and N inputs on microbial functional profiles by targeting the P cycling and the regulation of P availability in agroecosystems.

To date, most findings from natural forests, short-term field trials or pot experiments have revealed that low-P conditions facilitate microbial P immobilization [5, 20, 21]. For instance, Spohn et al. [20] reported that microbial P-uptake process was faster from relatively P-deficient than P-adequate soils planted with young beech trees. Bergkemper et al. [5] showed the lower functional potential of genes coding for P-uptake and transport (*pst* genes) in P-rich than P-poor forest soils. The abundance of the gene coding for alkaline phosphatase (*phoD*) was decreased by P fertilization after 14 weeks of grass growth [21]. With the interplay of N and P turnover, N input also affects P-transformation processes in soil because microorganisms have to maintain stable N:P stoichiometry for their growth. For example, elevated N addition stimulated the activity of phosphatase enzymes (e.g., phosphomonoesterase and acid phosphatase) or increased the abundance of genes involved in P-solubilization [22, 23]. However, long-term N inputs can decrease soil pH, restrain microbial growth and alter microbial community composition [24], and hence decrease P-solubilizing capacity. This has been partially supported by a meta-analysis showing no change in soil labile P or microbial biomass in terrestrial ecosystems following N addition [25]. In addition, Ragot et al. [26] reported that the dominant bacterial groups containing genes coding for alkaline phosphatase were mainly affected by soil pH, indicating that pH change caused by N input may alter microbial P-transformation communities. Thus, understanding the distribution, abundance, and connection of genes associated with P cycling under long-term N and P inputs and the underlying mechanisms will provide a novel insight into the P cycling in sustainable agroecosystems from a microbial gene perspective.

This study explored the long-term effects of frequent inputs of two key nutrients, N and P, on microbial P-transformation communities by metagenomics. Soil samples were collected from four long-term experimental sites across China, with different soil types, crop types, and climatic conditions. Our findings will provide a good reference for regulating P cycling in sustainable agroecosystems if consistent responses of gene composition to N and P inputs are observed at the four long-term experimental sites. Our aims were to (1) investigate the responses of the genes involved in inorganic P-solubilization and organic P-mineralization, P-uptake and transport, and P-starvation response regulation to long-term N or P inputs; (2) find the dominant influential factor that regulates microbial genes involved in P-transformation under N or P inputs; and (3) identify the dominant P-solubilizing and mineralizing microorganisms which are highly responsive to N or P inputs. We hypothesized that (1) long-term P input would increase microbial P-uptake and assimilation by directly supplying P for microorganisms, and (2) long-term N input would increase microbial P-solubilization and mineralization due to the increased microbial demand for available P, based on microbial N:P stoichiometric homeostasis.

## Methods and materials

### Site description and agricultural practices

Soil samples were collected from four experimental sites with long-term N and P inputs across China, including Key Field Observation Station of Lateritic Red Soil Eco-environment in Guangzhou (GZ), Guangdong Province (113° 26′ E, 23° 23′ N), Jiangxi Institute of Red Soil in Jinxian (JX), Jiangxi Province (116° 20′ E, 28° 15′ N), Shenyang Agricultural University, Shenyang (SY), Liaoning Province (123° 34′ E, 41° 49′ N) and the Black Soil Ecological Experimental Station in Harbin (HB), Heilongjiang province (126° 35′ E, 45° 40′ N) (Table S1).

The GZ site was established in 2011 on a Red soil (Ultisol) with a cabbage–cabbage–eggplant rotation. The site had three treatments: (1) N + P + K + M (manure) input; (2) N + K + M input; and (3) P + K + M input. The application rates of N, K, P, and manure are described in Table S1. The JX site, established in 1986, had grown continuous maize on a Red soil (Ultisol). This site had three treatments: (1) control (CK), no chemical nutrient applied; (2) N input alone (N), with 120 kg N ha^−1^ y^−1^ as urea; and (3) P input alone (P), with 26.2 kg P ha^−1^ y^−1^ as calcium magnesium phosphate. The SY site, established in 1987, had a Brown soil (Alfisol) with continuous maize cropping. This site had three treatments: (1) control (CK), no chemical nutrient applied; (2) N input alone (N), with 150 kg N ha^−1^ y^−1^ as urea; and (3) N + P input (NP), with 150 kg N ha^−1^ y^−1^ as urea and 29.5 kg P ha^−1^ y^−1^ as ammonium dihydrogen phosphate. The HB site, established in 1979, had a Black soil (Mollisol) with a maize–soybean–wheat rotation. This site had three treatments: (1) control (CK), no chemical nutrient applied; and (2) N input alone (N) at 150, 75, and 150 kg N ha^−1^ y^−1^ as urea for wheat, soybean, and maize, respectively; and (3) P input alone (P), with application rates of 32.7, 65.5, and 32.7 kg P ha^−1^ y^−1^ as calcium superphosphate and diammonium phosphate for wheat, soybean, and maize, respectively. These field experiments had a randomized block design with each treatment receiving three true replicates. Full details of the experimental sites are given in Table S1.

Surface soils (0–10 cm) from the four sites were sampled in May, 2017, and the information about crop type, growth stage, and fertilization at sampling time was presented in Table S1. We collected samples from three plots (three replicates) for each treatment per site. In each plot, seven soil cores were collected following an “S” sampling pattern, and then pooled to form a composite sample. The soils were then sieved through 2 mm after removal of plant residues and stones. Each soil sample was divided into two parts; one was air-dried, ball-milled, sieved to <0.15 mm, and homogenized for chemical analyses, and the other stored at −20 °C prior to DNA extraction. In total, four experimental sites with three treatments at each site were used in this study. Each site included two comparisons (Table S1). The first comparison was between two treatments without P (−P) and with P input (+P). The other comparison was between two treatments without N (−N) and with N applied (+N). Thus, four nutrient input groups were used: (1) −P, where P was not applied; (2) +P, where P was applied; (3) −N, where N was not applied; and (4) +N, where N fertilizer was applied to the four sites.

### Soil chemical analyses

Soil pH was measured after shaking soil in a water suspension of 1:2.5 soil:water ratio for 30 min. Soil microbial biomass P was determined using the chloroform fumigation-extraction method [27]. Concentrations of total N in soil were determined using a Flash EA 1112 elemental analyzer (Thermo Scientific, USA). Concentrations of total P in soil were determined using the molybdenum blue colorimetric method after digestion with H_2_SO_4_–HClO_4_. Soil N:P was calculated by dividing the concentrations of total N by total P. Available P in soils with pH below 7.0 was extracted using 0.5 mol L^−1^ HCl and 0.025 mol L^−1^ H_2_SO_4_, while that above pH 7.0 was extracted with 0.5 mol L^−1^ NaHCO_3_, pH 8.5 [27]. The extracted P was measured as above. Soil P fractionation, including Al–P, Fe–P, Ca–P, and O–P (occluded-P), was carried out according to Hedley et al. [28]. The total inorganic P content was calculated as the sum of the concentrations of Al–P, Fe–P, Ca–P, and O–P.

### DNA extraction and sequencing

Soil DNA was extracted using the FastDNA SPIN kit (MP Bio-medicals, Solon, OH, USA) following the manufacturer's protocol. Prepared DNA samples were then sent to Novogene Co., Ltd (Tianjin, China) for library preparation and shotgun metagenomics sequencing. The quality and concentration of DNA samples were monitored by agarose gel electrophoresis and Qubit^®^ 2.0 Flurometer. A total input amount of 1 μg DNA per sample was used for the library construction. The NEBNext^®^ Ultra™ DNA Library Prep Kit for Illumina (NEB, USA) was used to generate sequencing libraries following manufacturer’s instructions. Barcodes were added to each sample to give the attribute for sequences. Sonication was used to fragment DNA sample to a size around 350 bp. Then, PCR amplification was conducted after the DNA fragments were end-polished, A-tailed, and ligated with the adapter. AMPure XP system was used for PCR product purification. Agilent 2100 Bioanalyzer was used to analyze the size distribution of DNA library and real-time PCR was used to quantify DNA libraries. A cBot Cluster Generation System was employed to cluster the barcoded samples, according to the manufacturer’s recommendations. At last, the samples were sequenced and paired-end reads were generated on the instrument NovaSeq 6000.

The adapters, which were ligated to the DNA during the library preparation, were removed from sequencing data using instrument built-in analysis software. Readfq.v8_meta (https://github.com/NovogeneMicro/readfq.v8_meta) was then used to control the quality of raw data. First, the reads that contain low-quality bases above a certain portion were entirely removed (quality threshold value was ≤38; length was set up to 40 bp). Second, the reads containing N base that reached a certain percentage were removed (length was set up to 10 bp) [29]. Third, the reads which overlap with the adapter above a certain portion (length was set up to 15 bp) were removed. Fourth, the artificial duplicate reads were not removed [30, 31]. Overall, a total of ~1.3 billion paired-end reads (read length = 150 bp) were produced, ranging from ~33.6 to 38.1 million reads per sample (Table S2).

The filtered reads (*fastq* formats) were assembled using the MEGAHIT via *de Bruijn* graph and with the minimum k-mer size of 21 [32]. Prodigal (a gene prediction algorithm) was then used to predict the protein-coding gene with default settings [33]. Diamond software was used to blast predicted genes against the nonredundant protein sequences database of NCBI (https://www.ncbi.nlm.nih.gov/) with default settings [34], using BLASTP (best hit with *E* < 0.001). Functional annotation was conducted by aligning sequencing reads against KEGG database (Release 84.1) [35] using MEGAN6 software (Version 6.11.7) [36] with the parameter setting of blastp [34] based on the LCA algorithm. Finally, the gene read numbers for each sample were normalized based on median read number. The relative abundances (percentage) of genes were calculated related to the annotated reads and used for subsequent analyses.

The genes involved in soil microbial P-transformation were searched in the datasets based on previous publications [5, 8, 37, 38]. The genes involved in intracellular phosphatase production in microbial metabolic processes were excluded in this analysis as they do not typically participate in soil P cycling [5]. In total, 40 genes involved in P-transformation with their corresponding KO numbers were collected. We classified these genes into three categories according to their functional roles in soil P cycling: (1) genes involved in microbial P-starvation response regulation, a global regulation network that controls genes involved in P-uptake and P-solubilization and mineralization, (2) genes involved in microbial inorganic P-solubilization and organic P-mineralization; and (3) genes involved in microbial P-uptake and transport that control the immobilization of soil P into the microbial biomass. The names, functions, and classifications of the genes associated with P cycling are shown in Table S3. In addition, to obtain the taxonomic assignments of specific genes, sequences of predicted genes from the KEGG database were assigned against the NCBI nonredundant protein sequences database by using Diamond and MEGAN6 (details in Supporting Information). All sequences were deposited in the European Nucleotide Archive under the study accession number: ERP109781.

### Statistical analysis

Analysis of similarities (ANOSIM) [39] was conducted to explore the similarities and differences in the gene composition for P-transformation using the “vegan” R package [40], and then presented by nonmetric multidimensional scaling plots (NMDS) using the Bray–Curtis dissimilarity matrix. Distance-based linear modeling (DistLM) was performed using PRIMER 7 (Plymouth Routines in Multivariate Ecological Research Statistical Software, v7.0.13) to investigate the relationships between microbial gene composition for P-transformation and environmental factors (i.e., pH and N:P ratio) with *p* values adjusted by Bonferroni-correction [41, 42]. Significant differences in the relative abundance of genes involved in P-transformation between the –P and +P treatments, and between the –N and +N treatments were determined by two-way analysis of variance (ANOVA) with *p* values adjusted by Bonferroni-correction [43], after the normality of residues and homogeneity of variance were checked using Shapiro–Wilk and levene test, respectively, conducted using SPSS 18.0. One-way ANOVA was performed to investigate the treatment effect on soil chemical properties (Fig. S1). The relationships between relative abundances of genes and soil chemical properties (e.g., pH and N:P ratio) were tested using Spearman’s Rank correlations. Gene co-occurrence networks for the −P, +P, −N, and +N treatments were structured based on the Spearman’s correlation matrix with the threshold value identified by random matrix theory and the *p* values of correlation was <0.01 adjusted with the false discovery rate method [44, 45]. The topological parameters of resulting networks were calculated using the igraph package [46]. Networks were then visualized with an interactive platform Cytoscape [47]. Structural equation model (SEM) was conducted to quantify the effects of N and P inputs on the soil microbial gene composition for P-transformation. The maximum likelihood calculation was used to fit the covariance matrix to the model by using the IBM SPSS-Amos 26.0 with the Chi-square (*p* > 0.05) and root mean square error of approximation <0.05.

## Results

### Soil properties

Long-term P input increased both soil microbial biomass P by 7.4–44.5 mg kg^−1^ (*p* < 0.05) (except for the HB site), P availability by 33–163 mg kg^−1^ and total P concentration by 0.18–0.90 g kg^−1^ (*p* < 0.05), but did not affect soil pH and total N concentration (*p* > 0.05) at the four sites (Fig. S1). In comparison, long-term N input did not affect soil microbial biomass P (except for the HB site), total P concentration or total N concentration (*p* > 0.05), but decreased P availability by 2–610 mg kg^−1^ (*p* < 0.05) and pH by 0.40–1.57 units (*p* < 0.05) (Fig. S1).

### Microbial gene composition for P-transformation

**Fig. 1:**
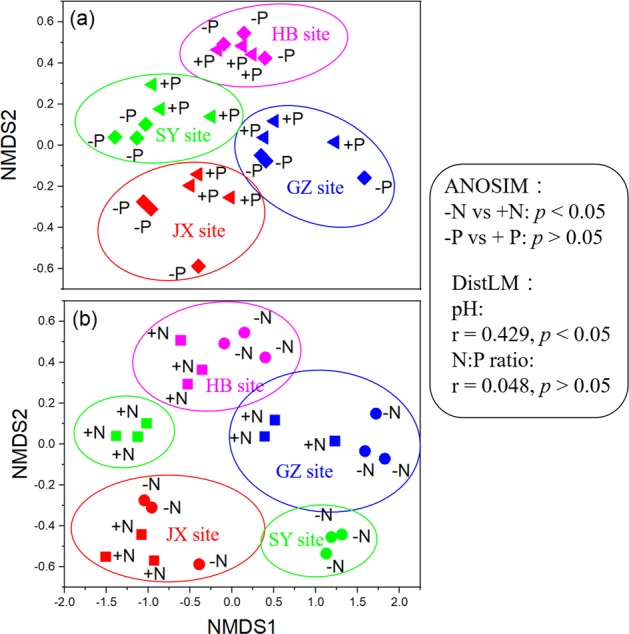
Nonmetric multidimensional scaling plots (NMDS) of microbial gene composition for P-transformation in soils with and without P or N input. ANOSIM showed significant differences (*p* < 0.05) in gene composition between the –N and +N treatments, and nonsignificant differences (*p* > 0.05) between the –P and +P treatments. DistLM indicates that soil pH had the closer correlations with gene composition compared with soil N:P ratio.

Long-term N input changed the composition of microbial genes involved in P-transformation between the four experimental sites (*p* < 0.05), while the effects of P input on the gene composition was not significant (Fig. 1). Soil pH and N:P ratio explained 43% (*p* < 0.05) and 4.8% (*p* > 0.05) of the variation in gene composition for P-transformation, respectively, shown by the DistLM analysis.

### Relative abundance of genes involved in P-transformation

**Fig. 2:**
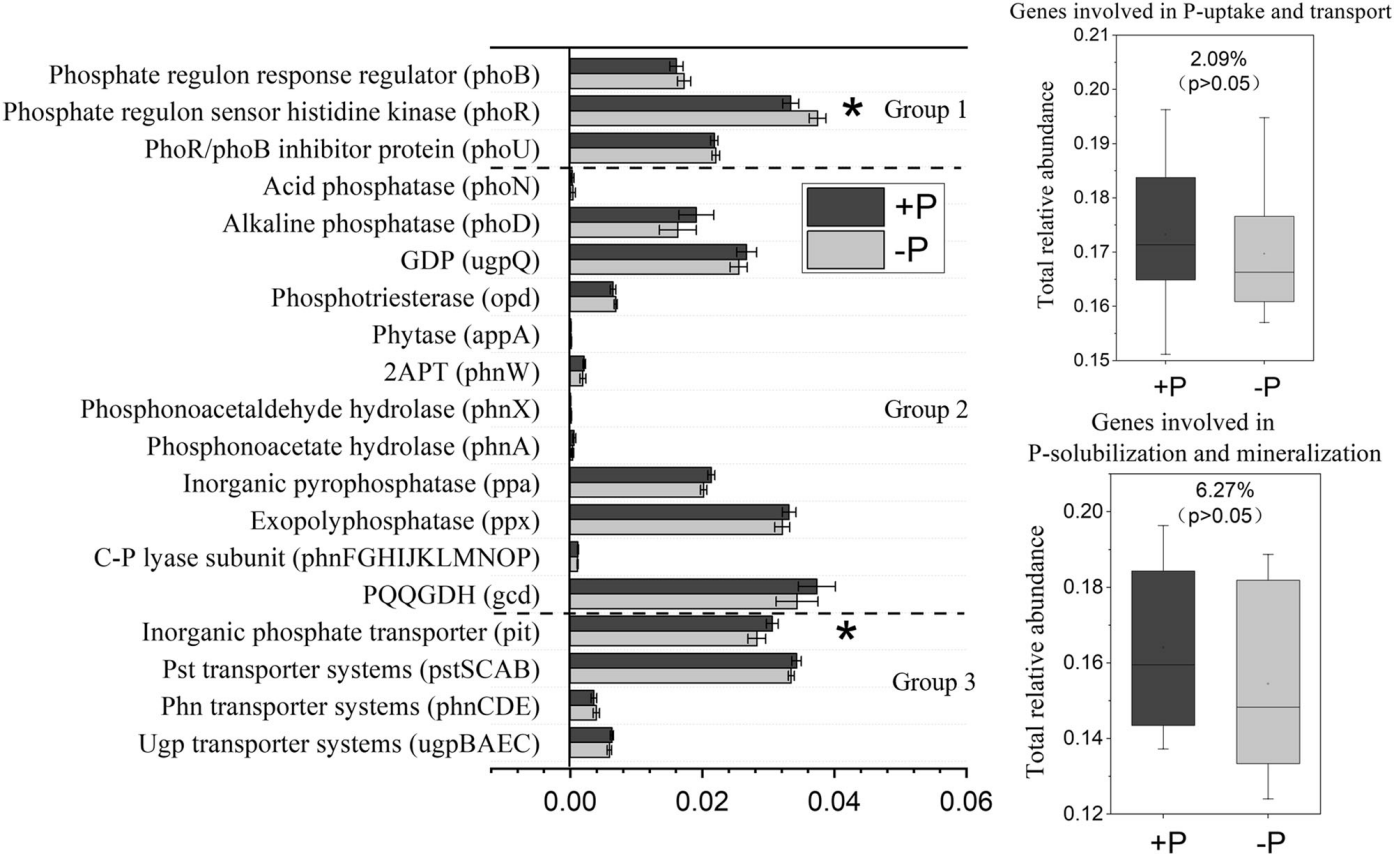
Relative abundance of representative genes responsible for microbial (1) P-starvation response regulation, (2) inorganic P-solubilization and organic P-mineralization, and (3) P-uptake and transport in soils with and without P input. The relative abundances of genes were calculated related to the annotated reads. Group 1, Genes coding for P-starvation response regulation; Group 2, Genes coding for inorganic P-solubilization and organic P-mineralization; Group 3, Genes coding for P-uptake and transport. Asterisk represents the significant effects of P input on the relative abundance of genes involved in P-transformation at *p* < 0.05. The effect of experimental site and the interactions between P input and site are presented in Table S4. 2APT 2-aminoethylphosphonate-pyruvate transaminase, GDP glycerophosphoryl diester phosphodiesterase, PQQGDH quinoprotein glucose dehydrogenase. Error bars are ±standard error. The values on the right boxes (i.e., 2.09 and 6.27%) indicating the shift in relative gene abundance in response to the P input, were calculated using the equation:$\frac{{{\mathrm{total}}\,{\mathrm{gene}}\,{\mathrm{relative}}\,{\mathrm{abundance}}\,{\mathrm{in}} +{\mathrm{P}}\,{\mathrm{treatments}}\,-\,{\mathrm{total}}\,{\mathrm{gene}}\,{\mathrm{relative}}\,{\mathrm{abundance}}\,{\mathrm{in}} -{\mathrm{P}}\,{\mathrm{treatments}}}}{{{\mathrm{total}}\,{\mathrm{gene}}\,{\mathrm{relative}}\,{\mathrm{abundance}}\,{\mathrm{in}} -{\mathrm{P}}\,{\mathrm{treatments}}}}.$ The relative abundance of *ugp* transporter systems was calculated as the average abundances of gene *ugpB*, *ugpA*, *ugpE*, and *ugpC*; the *phn* transporter systems was calculated as the average abundances of gene *phnC*, *phnE*, and *phnD*; the *pst* transporter systems was calculated as the average abundances of gene *pstB*, *pstC*, *pstA*, and *pstS*; the C-P lyase subunit was calculated as the average abundances of gene *phnF*, *phnG*, *phnH*, *phnI*, *phnJ*, *phnK*, *phnL*, *phnM*, *phnN*, *phnO*, and *phnP*.

**Fig. 3:**
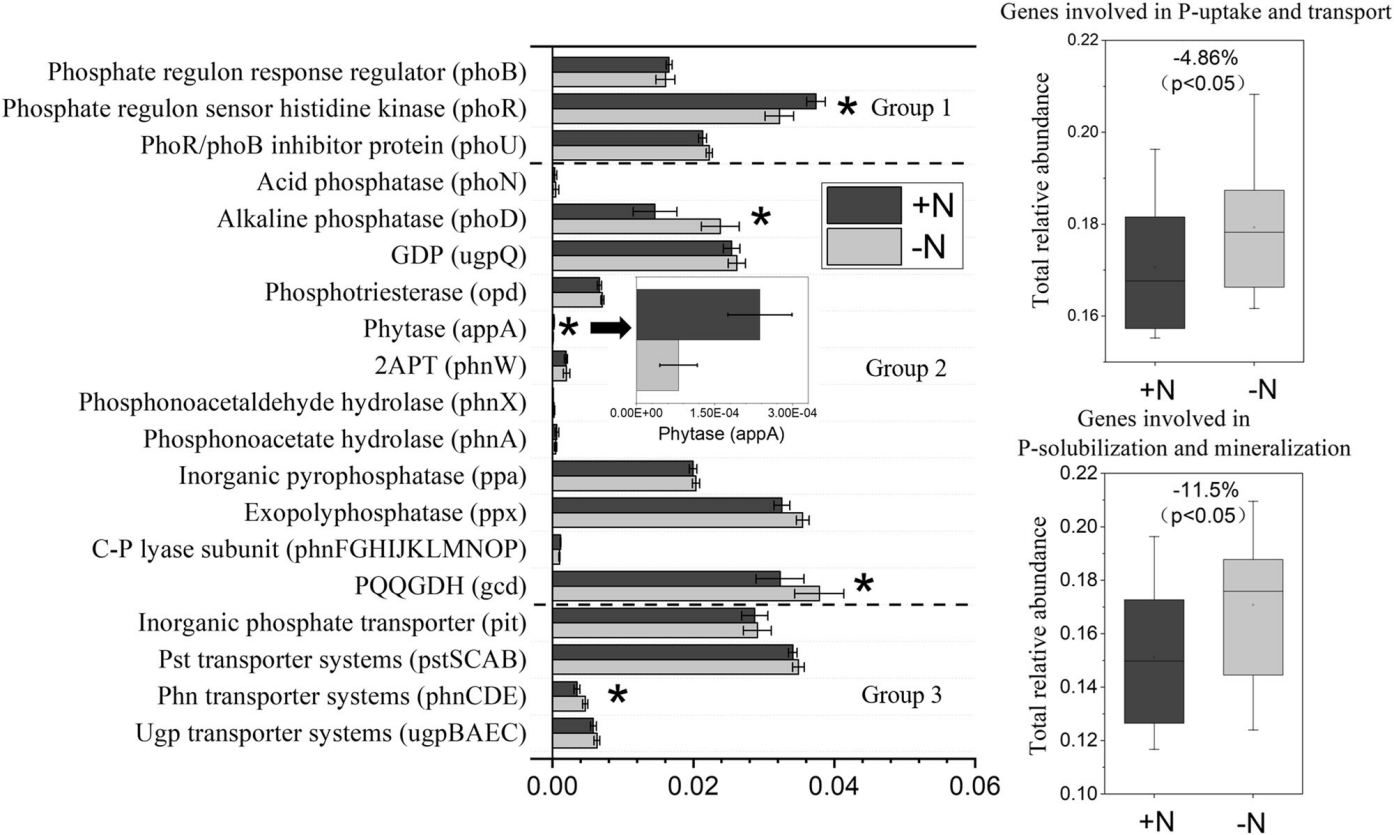
Relative abundance of representative genes responsible for microbial (1) P-starvation response regulation, (2) inorganic P-solubilization and organic P-mineralization, and (3) P-uptake and transport in soils with and without N input. The relative abundances of genes were calculated related to the annotated reads. Group 1, Genes coding for P-starvation response regulation; Group 2, Genes coding for inorganic P-solubilization and organic P-mineralization; Group 3, Genes coding for P-uptake and transport. Asterisk represents the significant effects of N input on the relative abundance of genes involved in P-transformation at *p* < 0.05. The effect of experimental site and the interactions between N input and site are presented in Table S5. 2APT 2-aminoethylphosphonate-pyruvate transaminase, GDP glycerophosphoryl diester phosphodiesterase, PQQGDH quinoprotein glucose dehydrogenase. Error bars are ±standard error. The relative abundances of genes were calculated related to the annotated reads. The values on the right boxes (i.e., −4.86 and −11.5%), indicating the shift in relative gene abundance in response to the N input, were calculated using the equation:$\frac{{{\mathrm{total}}\,{\mathrm{gene}}\,{\mathrm{relative}}\,{\mathrm{abundance}}\,{\mathrm{in}}\, +{\mathrm{N}}\,{\mathrm{treatments}}\,-\,{\mathrm{total}}\,{\mathrm{gene}}\,{\mathrm{relative}}\,{\mathrm{abundance}}\,{\mathrm{in}} -{\mathrm{N}}\,{\mathrm{treatments}}}}{{{\mathrm{total}}\,{\mathrm{gene}}\,{\mathrm{relative}}\,{\mathrm{abundance}}\,{\mathrm{in}} -{\mathrm{N}}\,{\mathrm{treatments}}}}.$ The relative abundance of *ugp* transporter systems was calculated as the average abundances of gene *ugpB*, *ugpA*, *ugpE*, and *ugpC*; the *phn* transporter systems was calculated as the average abundances of gene *phnC*, *phnE*, and *phnD*; the *pst* transporter systems was calculated as the average abundances of gene *pstB*, *pstC*, *pstA*, and *pstS*; the C-P lyase subunit was calculated as the average abundances of gene *phnF*, *phnG*, *phnH*, *phnI*, *phnJ*, *phnK*, *phnL*, *phnM*,* phnN*, *phnO*, and *phnP*.

Long-term P input had no effect on the total relative abundance of the genes involved in inorganic P-solubilization and organic P-mineralization and those involved in P-uptake and transport (*p* > 0.05) (Fig. 2), while long-term N input decreased the total relative abundance of the genes involved in inorganic P-solubilization and organic P-mineralization by 11.5% and the genes involved in P-uptake and transport by 4.9% (*p* *<* 0.05) (Fig. 3). With genes involved in P-starvation response regulation, long-term P input decreased the relative abundances of the *phoR* gene by 10.7% (*p* < 0.05) (Fig. 2). In contrast, long-term N input increased the relative abundance of the *phoR* gene by 16.1% (*p* < 0.05) (Fig. 3).

With individual genes coding for P-solubilization and mineralization, P input did not decrease the relative abundance of genes coding for alkaline phosphatase and PQQGDH (*p* *>* 0.05) (Fig. 2). Nitrogen input decreased the relative abundance of genes coding for alkaline phosphatase and PQQGDH (*p* *<* 0.05) but increased that of the genes coding for phytase (*appA*) (Fig. 3). With individual genes coding for P-transport and uptake, P input increased the relative abundance of the low-affinity inorganic phosphate transporter (*pit*) (*p* *<* 0.05) (Fig. 2), while neither N nor P input affected the relative abundance of the high-affinity phosphate-specific transporter (*pstSCAB*) (*p* *>* 0.05) (Figs. 2 and 3). Moreover, the interactive effects of P input and experimental site were not significant (*p* > 0.05) for the relative abundances of genes involved in P-transformation (Table S4). However, the significant interactive effects of N input and experimental site (*p* < 0.05) were observed in the relative abundances of some specific genes coding for C-P lyase subunit, phosphotriesterase, alkaline phosphatase, and some others (Table S5).

### Co-occurrence of microbial genes involved in P-transformation

**Fig. 4:**
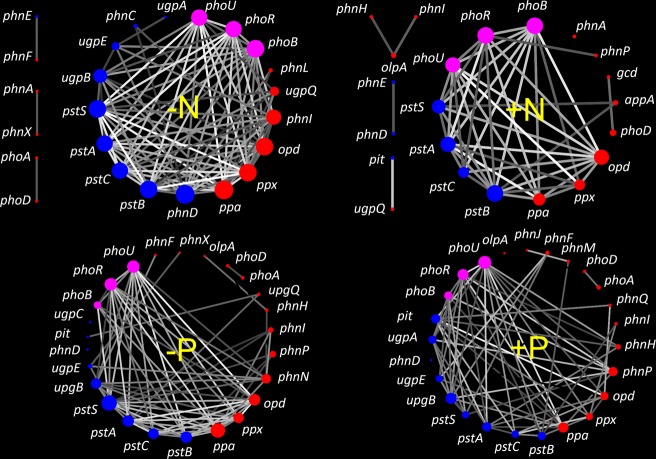
Occurrence networks of genes involved in P-transformation in soils with and without N or P input. The node size indicates the strength of the connections with other nodes (genes). Nodes with purple, blue, and red colors represent the genes involved in P-starvation response regulation, P-uptake and transport and inorganic P-solubilization and organic P-mineralization, respectively. The line color indicates the correlation coefficient of association for edges. The basic topological properties and their explanations of the four networks are shown in Table S4.

Long-term P input decreased the network topological parameters, i.e., average degree, graph density, and average clustering coefficient, which represented the degree of the network complexity, by 0.32, 0.013, and 0.089, respectively (Fig. 4 and Table S6). However, N input led to a stronger decrease in these parameters by 3.1, 0.12, and 0.13, respectively (Fig. 4 and Table S6). P input apparently decreased the node degree (i.e., connections between genes) for the high-affinity phosphate-specific transporters *pstSCAB* and *phoR*, but increased it with the low-affinity inorganic phosphate transporter *pit*. In comparison, N input apparently decreased the node degree for the genes involved in P-starvation response regulation (*phoR, phoB*, and *phoU)*, genes involved in P-uptake and transport (e.g., *pstSCAB*, *phnCD*, and *ugpABE*) and genes involved in organic P-solubilization (e.g., *ppa, opd, ppx*, *phnI*, and *ugpQ*).

### Taxonomic assignments of typical genes involved in P-solubilization and mineralization

**Fig. 5:**
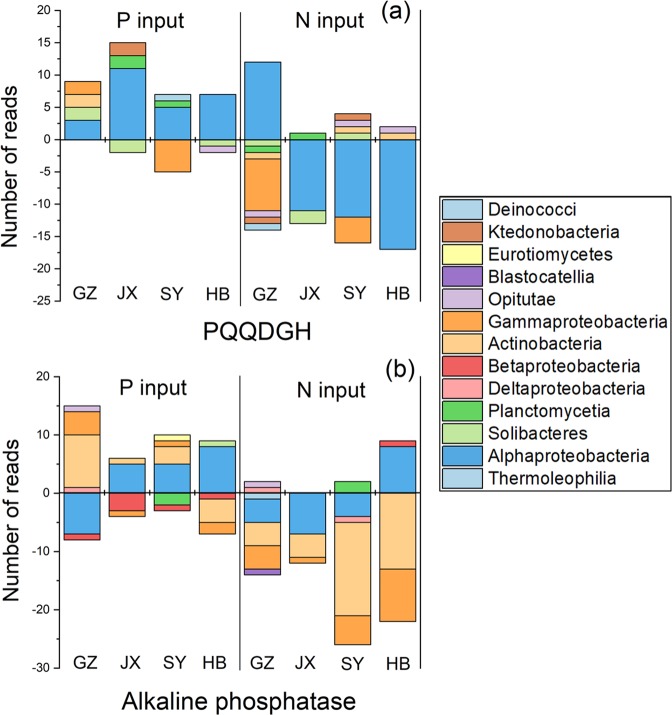
Changes in the abundances of taxonomic assignments of genes coding for PQQDGH (*gcd*) and alkaline phosphatase (*phoA* and *phoD*) synthesis at the class level in soils with N or P input history at four experimental sites. The values on the *Y* axis were calculated by subtracting normalized read numbers in the –N and –P treatments from those in the +N and +P treatments, respectively.

With taxa containing the gene coding for PQQDGH, long-term P input increased the assigned sequence number of *Alphaproteobacteria*, while N input decreased the number of *Alphaproteobacteria* (except for the GZ site) (Fig. 5a). With taxa containing genes coding for alkaline phosphatase, long-term P input decreased the assigned sequence number of *Betaproteobacteria* but increased that of *Actinobacteria* (except the HB site) and *Alphaproteobacteria* (except the GZ site) (Fig. 5b). In comparison, N input decreased the assigned sequence number of *Actinobacteria*, *Gammaproteobacteria*, and *Alphaproteobacteria* (except the HB site) (Fig. 5b). At the order level of the taxonomic assignments of genes involved in overall P-solubilization and mineralization, P input, in most cases, decreased the abundance of *Rhodospirillales* and *Acidobacteriales*, and increased that of *Rhizobiales* (Fig. S2). Nitrogen input mainly decreased the abundance of *Rhizobiales*, but increased those of *Rhodospirillales* and *Acidobacteriales* (Fig. S2).

### Influential factors on microbial gene composition for P-transformation

The soil N:P ratio was negatively (*p* < 0.05) correlated with the relative abundances of the genes coding for P-uptake and transport, i.e., *pstSCAB* (with a correlation coefficient of −0.41), *ugpABCE* (−0.37), and *phnCDE* (−0.24) (Fig. S3), and these genes positively correlated with the concentrations of individual P fractions (e.g., Al–P, Fe–P, O–P, and Ca–P) (Table S7). The soil pH was correlated positively (*p* < 0.05) with the relative abundances of genes responsible for PQQGDH (0.86) and alkaline phosphatase (0.92), and negatively (*p* < 0.05) with those of genes responsible for synthesis of acid phosphatase (−0.66) and phytase (−0.42) (Fig. S3). The gene coding for PQQGDH was positively correlated with concentrations of P fractions (Table S7), while the *phoR* gene (−0.65) was negatively correlated with soil pH (*p* < 0.05) (Fig. S3).

**Fig. 6:**
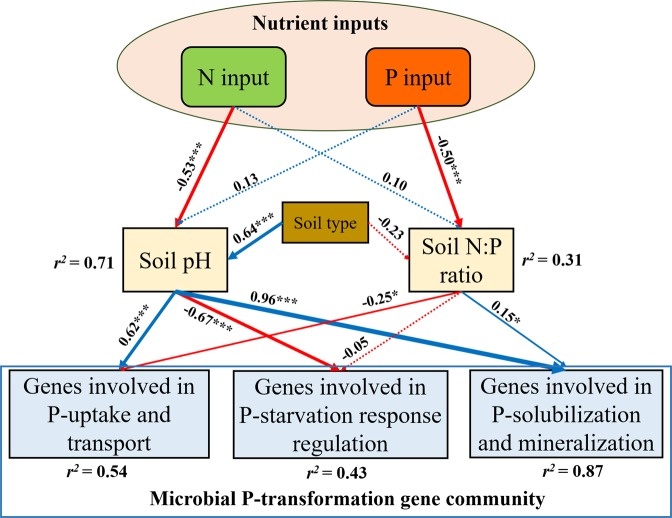
Structural equation model (SEM) illustrating how the N and P inputs influenced the gene composition for microbial P-transformation by changing soil pH and N:P stoichiometry. Solid and dashed arrows represent the significant and nonsignificant relationships between different variables. Adjacent values near the arrows indicate path coefficients. *r*^2^ values indicate the proportion of variance explained by each variable. Significance levels are denoted with *p < 0.05, **p < 0.01, and ***p < 0.001.

Based on the SEM, N inputs significantly affected the genes involved in microbial P-transformation by decreasing soil pH, while P inputs significantly affected the genes involved in microbial P-transformation by decreasing soil N:P ratio (Fig. 6). The decrease in soil pH induced by N inputs had positive effects on the genes involved in inorganic P-solubilization and organic P-mineralization (with an estimated value of 0.96, *p* < 0.05) and the genes involved in P-uptake and transport (0.62, *p* < 0.05), and had negative effects on the genes involved in P-starvation response regulation (−0.67, *p* < 0.05) (Fig. 6). The decreases in soil N:P ratio induced by P inputs had positive effects on the genes involved in inorganic P-solubilization and organic P- mineralization (with an estimated value of 0.15, *p* < 0.05), and had negative effects on the genes involved in P-uptake and transport (−0.25, *p* < 0.05) (Fig. 6).

## Discussion

### Effects of nutrient inputs on genes involved in P-transformation

#### Effects of P inputs on genes involved in P-transformation

In general, microbial P-assimilating capacity is mainly controlled by the *phoR*/*phoB* two-component system, high-affinity phosphate-specific transporters (*pstSCAB*) and low-affinity inorganic phosphate transporters (*pit*). These genes are mainly influenced by the environmental P supply [8]. The two-component system *phoR* and *phoB* regulates the expression of *pst* transporter systems (*pstSCAB*) to effectively utilize alternative P sources under low-P conditions [48, 49]. Our results, showing the increased relative abundance of *phoR* without P input (Fig. 2), agree with previous findings that low-P condition activated the *phoR* gene in *Escherichia coli* [8]. Although the relative abundance of *phoR* gene was enhanced in soils without P input, the high-affinity *pst* transporters did not respond correspondently. This result indicates that the connections (e.g., signal transduction pathway) between *phoR* and *pst* transporters were blocked to some extent (Fig. 4). In addition, the *pst* transporters and *phoR* may require the different soil P levels to respond as the heterogeneous P background levels in the no-P treatment at different trial locations or even within a same location (Fig. S1). Thus, we assume that the high-affinity *pst* transporters may respond under extremely low soil P conditions. Instead, the low-affinity inorganic phosphate transporter (*pit*) significantly increased with P input, leading to the increased microbial biomass P, and hence would play a more important role in microbial mineral P-assimilation when P is added in agroecosystems. This result is also consistent with the increased microbial biomass after mineral P addition [50] and the positive correlation between microbial biomass N:P and soil N:P ratios [51]. The decrease in the node degrees (i.e., gene connection) of *pstSCAB* genes and the increase in the node degree of *pit* gene (Fig. 4) also support this assumption. In contrast, the increased potentials of *pstSCAB* and no changes in *pit* were observed under low-P status in natural forest soils [5]. Such discrepancies are attributed to the different managements of the two ecosystem types. Natural forest soils are stable ecosystems with minimal anthropological disturbances where microbial properties are close to the reported phenotype of specific strains (e.g., *E. coli*), while agroecosystems usually receive high P inputs, which may result in irreversible alterations in microbial communities with time.

Previous estimates indicate that the turnover time of total soil microbial biomass is several months under laboratory conditions, suggesting that microbial biomass P can be considered as a pool of potentially plant-available P. In comparison, in the field with differences in soil type, crop type, temperature, and moisture, the biomass turnover time will vary by several fold and the competition of microbes for soil available P with plants would be possible and complicated [52, 53]. For instance, P input increased microbial biomass P at the SY and JX sites grown with maize but not at the HB site where soybean had grown (Table S1 and Fig. S1). Thus, microorganisms may temporarily compete for the available P with plants, depending on plant species, especially when P application and plant growth occur simultaneously. The extent of such competition and associated mechanisms deserve further investigations.

With microbial inorganic P-solubilization, the *gcd* gene comprising the glucose dehydrogenase (i.e., *gdh* gene) and the cofactor pyrroloquinoline quinone (i.e., *pqq* gene), regulates the solubilization of unavailable mineral P including Ca phosphates, hydroxyapatite, and some rock phosphate [54]. The mineralization of soil organic P is mainly attributed to the genes coding for alkaline phosphatases, e.g., *phoA* and *phoD* genes. These genes catalyze the hydrolysis of the two main organic P fractions in soils, i.e., phosphomonoesters and phosphodiesters [55], and have received considerable attention due to their high catalytic capacity and unique genetic properties [26, 56, 57]. Our study reveals that the lack of P input did not increase the abundance of the gene coding for PQQGDH compared with P addition (Fig. 2), which is inconsistent with the significantly higher abundance of the *pqq* gene in paddy soils with lower P inputs [58] and the increased expression and activity of the *pqq* gene under P-limiting conditions [59]. Similar to the *gcd* gene, the functional potentials of genes coding for alkaline phosphatase and other organic P-mineralizing enzymes were not enhanced by the lack of P inputs (Fig. 2). This contradicts the classical view that low-P condition favors the synthesis of phosphatases [9]. Under low-P conditions, microorganisms require extra energy to obtain P by regulating the gene involved in P-starvation response regulation (i.e., *phoR*) [4, 8] and enhancing release of phosphatases to mineralize soil organic P. In contrast, we only observed consistently higher abundances of the gene *phoR* in the −P soils compared with the +P soils (Fig. 2), in line with the findings of Hsieh and Wanner [8], but there were no increases in the abundance of the genes either coding for mineral P-solubilizing enzymes or organic P-mineralizing enzymes (Fig. 2). Some recent studies support our findings. For example, there was no difference in the functional potential of alkaline phosphatase between P-low and P-rich forest soils and the higher abundance of *gcd* gene was observed in P-rich forest soils [5]. In addition, the potential of alkaline phosphatase responded positively to the increasing rates of manure P addition [60]. These discrepancies between controlled-environment and field studies can be explained as follows. First, the functional traits of microbial organic P-mineralization have been mostly reported in specific microbial species grown in vitro [9], while they may behavior differently in the field [61]. Second, long-term low-P conditions (i.e., without P inputs for years) may induce irreversible changes to the microbial community, switching organic P-mineralizing microorganisms to a relatively inactive and dormant state. However, these microorganisms, when living under short-term stress of low P, can remain active. Shotgun metagenomics used in our study provides more comprehensive data that include both culturable and unculturable organic P-mineralizing microorganisms [62].

#### Effects of N inputs on genes involved in P-transformation

In general, based on the N:P stoichiometry, increasing N inputs enhances the microbial demand for inorganic P [63]. The capacity of microbial P-assimilation and P-solubilization and mineralization increases to obtain more inorganic P for microbial growth. However, our study shows that N input only increased the relative abundance of *phoR* gene, but had no effects on microbial biomass P in soil and the relative abundances of the high-affinity phosphate-specific transporters (*pstSCAB*) and low-affinity inorganic phosphate transporters (*pit*) (Fig. S1 and Fig. 3). This indicates that the functional potential of genes involved in P-uptake and transport and the corresponding microbial P-assimilation processes were still limited even though the functional potential of *phoR* enhanced under N input. In addition, N inputs weakened the roles of some key genes involved in P response regulation and transporter (e.g., *phoR* and *pstSCAB*) in the whole gene network (Fig. 4), which again reflects the low microbial P-assimilation capacity. Provided that soil pH had significant effects on *phoR* and genes involved in P-uptake and transport (Fig. 6), further investigations should be focused on whether the responses of these genes to N input are resulted directly from N supply, indirectly from associated pH change or both.

Our study has also demonstrated that N input decreased the relative abundance of the gene coding for PQQGDH (*gcd*) (Fig. 3), contrasting with the classical view that soil inorganic-P availability is regulated by “N:P stoichiometry”. In addition, the relative abundance of *gcd* gene decreased with decreasing soil pH caused by N input (Fig. S3), indicating that soil acidification plays a more important role in determining the potential of mineral P-solubilization than N:P stoichiometry. As previously reported, the microbial capacity of organic P-mineralization increases when N addition enables the microbes to obtain more inorganic P [13, 50], while N input decreased the potential of alkaline phosphatase in our study. As shown by the highly significant positive correlations between the relative abundance of genes coding for alkaline phosphatase and soil pH (Fig. S3), we propose that the low pH caused by long-term N input had a greater influence on the potential mineralization of phosphomonoesters and phosphodiesters than the N effect per se (i.e., N:P stoichiometry), as short-term or in vitro experiments with N addition often do not induce a large pH decrease. This agrees with previous findings that soil pH was one of the dominant factors influencing the composition of the alkaline phosphatase-synthesizing community in grassland [64] and between three different systems i.e., arable, forest, and grassland [26].

However, N input significantly increased the relative abundance of genes coding for phytase that can degrade phytate, which forms a large fraction of soil organic P, while the relative abundance of these genes negatively correlated with soil pH (Fig. S3). The increased potential of phytase following N input in our study could be attributed to: (1) N:P stoichiometry, i.e., an increased microbial demand for inorganic P, leading to an increased capacity for organic P-mineralization [13, 50]; and (2) the effects of low pH caused by N input, as indicated by the negative correlation between soil pH and gene abundance (Fig. S3). Therefore, the differential responses of the genes coding for alkaline phosphatases and those coding for phytase to N input indicate that soil pH and N:P stoichiometry both play important roles in microbial organic P-mineralization, while the contribution of pH was likely greater than that of N:P stoichiometry.

Overall, the decreased total potential of genes involved in P-solubilization and mineralization under long-term N inputs (Fig. 3) and the path coefficient of 0.96 from soil pH (Fig. 6) indicate that soil pH decrease was the dominant factor for the regulation of microbial P-solubilizing and mineralizing capacity and soil P availability. One explanation is that microbial abundances and communities are highly responsive to pH changes in arable soils [24]. Moreover, the severe acidification caused by long-term N input may inhibit microbial growth and activity, and hence the activity of phosphatases. The weakened connections between the genes coding for phosphatase (red dots in Fig. 4) and *phoR* gene after N input may also partially explain the decreased total relative abundance of the genes involved in inorganic P-solubilization and organic P-mineralization, since the *phoR* gene regulates the genes coding for alkaline phosphatase [10]. Due to the limitation of DNA-based metagenomics, further analyses such as RNA-based or quantitative high-throughput sequencing are suggested to test how the gene expression or absolute gene abundances respond to long-term N input.

As experimental sites had different crop rotations, different fertilization regimes, soil types, and sampling times, the relative abundance of genes involved in P-transformation differ greatly across different sites (Table S4, S5). Here, we emphasize the nonsignificant interaction between P input and experimental site on the functional potentials of most genes involved in P-transformation (Table S4). However, the significant interaction between N input and experimental site was observed, indicating that the site differences sometimes alter the effect of N input (Table S5). For instance, the higher available P in the –N than the +N treatment at the GZ site but comparable microbial biomass P between −N and +N treatments were likely attributed to the effects of manure addition, since the manure with extremely high P content met the need of microorganisms for P in the +N treatment. Moreover, the higher microbial biomass P in the –N than the +N treatment at the HB site might be attributed to the plantation of soybean, which had fixed N. The N supplied by soybean likely increased the microbial demand for P and so the microbial biomass P.

### Effects of nutrient inputs on typical P-solubilizing and mineralizing microorganisms

Due to the relatively fast turnover of microbial biomass P, we detailed the taxonomic assignments of genes involved in inorganic P-solubilization and organic P-mineralization, particularly on the microorganisms containing genes coding for PQQDGH and alkaline phosphatases. These genes contribute greatly to soil inorganic P-solubilization and organic P-mineralization as frequently reported recently [26, 55, 62]. They are also regarded as the indicators of P-solubilizing and mineralizing capacity in a given microbial community [2, 65].

To date, evidence has consistently revealed the stimulated growth and increased abundance of copiotrophic microorganisms such as *Proteobacteria* following N and P inputs compared with oligotrophic microorganisms such as *Acidobacteria* [18, 66–68]. The life cycle of microorganisms, depending upon nutrient availability, accounts for this trend [14]. In contrast, N input in most cases decreased the abundance of P-solubilizing and mineralizing microorganisms such as *Gammaproteobacteria* and *Alphaproteobacteria* (Fig. 5), most of which are primarily from copiotrophic microorganisms. This result indicates that the growth of microorganisms containing genes coding for PQQDGH or alkaline phosphatase is not determined by N availability. The decreases in soil pH resulting from N input and the negative correlations between genes coding for PQQDGH/alkaline phosphatase and soil pH at the four sites indicate that pH is the dominant factor determining the activity and/or function of these microorganisms. Similarly, Ragot et al. [57] showed that pH was the principal determinant of the structure and composition of the *phoD*-harboring microbial community in a 20-year fertilization trial. The increases in the abundance of *Alphaproteobacteria* by P inputs in our study indicate the determinants of P availability (Fig. 5) to some typical microorganisms with *phoD*/*phoA* genes, and that the increased P supply favored the growth of copiotrophic microorganisms. Previous studies also showed that P inputs significantly favored the growth of the phyla containing *phoD*/*phoA* such as *Proteobacteria* [26, 69]. The increase and decrease in the abundance of *Actinobacteria* with P and N inputs, respectively, indicate that *Actinobacteria* have a trophic life-style close to copiotrophic microorganisms, and is responsive to pH change, but further investigations are required.

With the genes involved in total P-solubilization and mineralization at the order level, the *Rhizobiales* in the class *Alphaproteobacteria*, plant-growth-promoting bacteria, are usually more abundant in P-rich soils [1, 5], which is supported by their increased abundance following P input (Fig. S2). Instead, the abundance of heterotrophic *Rhodospirillales* (from *Alphaproteobacteria*), not widely reported as a P-solubilizing bacteria, increased by the lack of P inputs. Future studies should focus on the phenotype of this isolate for P-solubilizing and mineralizing processes. The growth inhibition of *Acidobacteriales* by high P inputs accords with *Acidobacteriales* being oligotrophic bacteria [14]. In addition, the increased abundance of *Acidobacteriales* with decreasing soil pH agrees with the previous finding of the dominance of *Acidobacteriales* in acid soils [24].

Our study also showed that the responses of some microorganisms to P or N inputs varied with the sampling sites. The increased abundance of copiotrophic *Alphaproteobacteria* at the GZ site but the decreased abundance in the other sites by N inputs could be related to the application of nutrient-rich manure in the GZ site. The various responses to P input between the four sites of the abundance of *Actinobacteria* presumably resulted from the effect of crop types and soil properties; soybean at the HB site with black soil properties might have an inhibitory effect on *Actinobacteria*. Albeit the effect of sampling site, both P-solubilizing and mineralizing microorganisms were generally responsive to nutrient inputs and/or soil pH decreases.

**Fig. 7:**
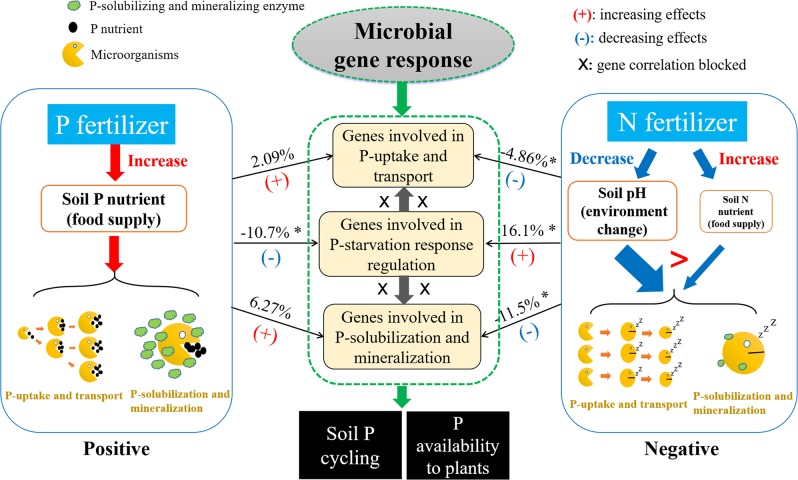
A summary diagram showing the responses of the genes involved in P-uptake and transport, inorganic P-solubilization and organic P-mineralization, and P-starvation response regulation to long-term N and P inputs. The asterisk above the solid arrows represent the average abundances of genes involved in P-transformation increased/decreased significantly (*p* < 0.05) with N or P input.

In conclusion, long-term N and P inputs have significant impacts on the genes involved in P-transformation via altering soil pH and microbial N:P stoichiometry. The details of these impacts are summarized in Fig. 7. We demonstrated that long-term P inputs increased microbial P immobilization, while long-term N inputs decreased the functional potential of microbial P-solubilization and mineralization by decreasing soil pH. These findings make new additions to the current concepts that low-P conditions facilitate microbial P immobilization, and that N:P stoichiometry regulates microbial P-solubilization and mineralization capacity. The study lays a foundation for manipulating specific microorganisms/genes to enhance P availability and regulate P cycling in agroecosystems. Furthermore, this study used soil samples collected at only one time point at four field sites differing in cropping systems, and there may be temporal variations in microbial functional profiles of P-transformation. Future studies may cover multiple sampling times and conduct designed field trials so that temporal variations and interactions between nutrient inputs and crop types can be elucidated in detail.

## Supplementary information

Supporting Information
