# Supplementary material for: Long-term nutrient inputs shift soil microbial functional profiles of phosphorus cycling in diverse agroecosystems
Source: ISME J. 2019 Dec 11;14(3):757–70. doi: 10.1038/s41396-019-0567-9 (PMC7031380; doi:10.1038/s41396-019-0567-9)
Supplement: Supplementary file 1 — Supporting Information [file 41396_2019_567_MOESM1_ESM.docx]

**Supporting Information**

**Long-term nutrient inputs shift soil microbial functional profiles of phosphorus cycling in diverse agro-ecosystems**

By Zhongmin Dai et al.

**Data processing script**

**1. Quality control**

**software: readfq.v8_meta**

readfq.v8_meta -z -f read.list -3 *.fq1 -4 *.fq2 -q 38, 40 -n 10 -l 15 1>*.out

read.list: *.raw.fq1.gz, *.raw.fq2.gz

**2. Sample assembly**

**software: Megahit v1.1.3**

megahit -1 *fq1.gz -2 *fq2.gz -m 0.85 -o ASSEMBLY/ -t $NUM_THREADS

**3.Gene prediction and abundance analysis**

**software: Prodigal v2.6.2**

prodigal -i ASSEMBLY/final.contig.fa -o /prodigal/Gene.* -a /prodigal/*.faa -p meta

**4. Taxonomy prediction and functional annotations**

**software: DIOMAND v0.9.19.120; Megan_Ultimate v 6.11.7**

diamond makedb --in nr.faa -d nr

diamond blastp -d nr.dmd -q /prodigal/*.faa --daa /prodigal/*.daa

megan/tools/daa-meganizer -i /prodigal/*.daa -a2t Database/prot_acc2tax-Mar2018X1.abin -a2kegg Database/acc2kegg-Dec2017X1-ue.abin

megan/tools/daa2info -i /prodigal/*.daa -r2c KEGG -n >05_kegg_read/KEGG_*.txt

megan/tools/daa2info -i /prodigal/*.daa -r2c Taxonomy -p -r >04_taxonomy_read/*.txt

**Table S1.** Site location, experimental duration, nutrient treatments and crop and fertilization details at the time of soil sampling at four long-term experimental sites.

| Site location | Longitude/ Latitude | Soil type | Crop type | N and P treatments  (Abbreviation) | Effect of  P input  (+P vs -P) | Effect of  N input  (+N vs -N) | Nutrient type  (annual application rate) | Experimental duration (years) | Crop & fertilization at sampling time |
| --- | --- | --- | --- | --- | --- | --- | --- | --- | --- |
| Guangzhou  (GZ) | 113^o^26’E  23^o^23’N | Ultisol | Cabbage-  cabbage-  eggplant rotation | **(1)** N+K+M (N)  **(2)** N+P+K+M (NP)  **(3)** P+K+M (P) | **(2) vs (1)** | **(2) vs (3)** | N: urea (720 kg N ha^-1^)  P: calcium superphosphate (107 kg P ha^-1^)  K: potassium chloride (427 kg K ha^-1^)  M: commercial manure (360 kg N ha^-1^; 291 kg P ha^-1^; 505 kg K ha^-1^) | 7 | Cabbage already harvested;  Fertilizers added about 1.5 months ago |
|  |  |  |  |  |  |  |  |  |  |
| Jinxian  (JX) | 116^o^20’E  28^o^15’N | Ultisol | Maize | **(1)** No input fertilizers (CK)  **(2)** N input alone (N)  **(3)** P input alone (P) | **(3) vs (1)** | **(2) vs (1)** | N: urea (120 kg N ha^-1^)  P: calcium magnesium phosphate (26.2 kg P ha^-1^) | 31 | Maize at early growth stage;  Fertilizers added about 1 month ago |
|  |  |  |  |  |  |  |  |  |  |
| Shenyang  (SY) | 123^o^34’E  41 ^o^49’N | Alfisol | Maize | **(1)** No input fertilizers (CK)  **(2)** N input alone (N)  **(3)** N+P input (NP) | **(3) vs (2)** | **(2) vs (1)** | N: urea (150 kg N ha^-1^)  P: ammonium dihydrogen phosphate (29.5 kg P ha^-1^) | 30 | Maize at early growth stage;  Fertilizers added about 1.5 months ago |
|  |  |  |  |  |  |  |  |  |  |
| Harbin  (HB) | 126°35′E  45°40′N | Mollisol | Maize-  soybean-  wheat rotation | **(1)** No input fertilizers (CK)  **(2)** N input alone (N)  **(3)** P input alone (P) | **(3) vs (1)** | **(2) vs (1)** | N: urea (150 kg N ha^-1^ for wheat, 75 kg N ha^-1^ for soybean and 150 kg N ha^-1^ for maize)  P: calcium superphosphate + diammonium phosphate (33 kg P ha^-1^ for wheat, 66 kg P ha^-1^ for soybean and 33 kg P ha^-1^ for maize) | 39 | Soybean at early growth stage;  Fertilizers added 1 month ago |

* The application rate of manure (M) at the Guangzhou site varies every year, and the average annual amount of nitrogen (N), phosphorus (P) and potassium (K) applied in three seasons are given.

**Table S2.** Summary of sequencing data from four long-term experimental sites. ADRs: artificial duplicate reads.

| **Sample** | **Raw data（Mbase）** | **Raw reads**  **(#)** | **Clean data**  **（Mbase）** | **Clean_Q20** | **Effective**  **(%)** | **ADRs（Mbase）** | **Percentage of ADRs**  **(%)** |
| --- | --- | --- | --- | --- | --- | --- | --- |
| JX-CK-1 | 5,430.00 | 36,200,024 | 5,415.32 | 96.88 | 99.73 | 369.63 | 6.8 |
| JX-CK-2 | 5,097.08 | 33,980,514 | 5,088.56 | 96.86 | 99.83 | 298.85 | 5.9 |
| JX-CK-3 | 5,686.32 | 37,908,828 | 5,671.37 | 96.95 | 99.74 | 397.71 | 7.0 |
| JX-N-1 | 5,074.19 | 33,827,966 | 5,067.79 | 97.06 | 99.87 | 394.19 | 7.8 |
| JX-N-2 | 5,144.46 | 34,296,408 | 5,135.34 | 96.91 | 99.82 | 386.19 | 7.5 |
| JX-N-3 | 5,086.07 | 33,907,140 | 5,072.79 | 95.35 | 99.74 | 196.63 | 3.9 |
| JX-P-1 | 5,407.71 | 36,051,388 | 5,392.09 | 97.22 | 99.71 | 424.60 | 7.9 |
| JX-P-2 | 5,247.88 | 34,985,842 | 5,233.26 | 97.22 | 99.72 | 382.35 | 7.3 |
| JX-P-3 | 5,199.00 | 34,660,020 | 5,171.87 | 95.82 | 99.48 | 268.51 | 5.2 |
| HB-CK-1 | 5,063.71 | 33,758,066 | 5,049.94 | 95.86 | 99.73 | 269.43 | 5.3 |
| HB-CK-2 | 5,548.18 | 36,987,890 | 5,537.90 | 95.53 | 99.82 | 267.63 | 4.8 |
| HB-CK-3 | 5,622.92 | 37,486,164 | 5,613.83 | 96.78 | 99.84 | 385.22 | 6.9 |
| HB-N-1 | 5,447.33 | 36,315,566 | 5,433.82 | 96.94 | 99.75 | 419.51 | 7.7 |
| HB-N-2 | 5,090.72 | 33,938,156 | 5,078.11 | 96.81 | 99.75 | 370.56 | 7.3 |
| HB-N-3 | 5,715.13 | 38,100,882 | 5,699.32 | 96.84 | 99.72 | 430.01 | 7.5 |
| HB-P-1 | 5,622.84 | 37,485,592 | 5,615.81 | 97.03 | 99.88 | 409.51 | 7.3 |
| HB-P-2 | 5,271.22 | 35,141,492 | 5,263.32 | 97.04 | 99.85 | 372.68 | 7.1 |
| HB-P-3 | 5,620.12 | 37,467,482 | 5,609.76 | 96.97 | 99.82 | 360.14 | 6.4 |
| SY-CK-1 | 5,137.62 | 34,250,812 | 5,124.34 | 95.38 | 99.74 | 220.58 | 4.3 |
| SY-CK-2 | 5,539.93 | 36,932,834 | 5,530.17 | 95.00 | 99.82 | 197.14 | 3.6 |
| SY-CK-3 | 5,363.40 | 35,756,002 | 5,348.92 | 95.05 | 99.73 | 193.60 | 3.6 |
| SY-N-1 | 5,348.64 | 35,657,592 | 5,334.11 | 96.74 | 99.73 | 345.35 | 6.5 |
| SY-N-2 | 5,401.57 | 36,010,436 | 5,375.32 | 94.98 | 99.51 | 227.01 | 4.2 |
| SY-N-3 | 5,673.59 | 37,823,938 | 5,659.75 | 96.65 | 99.76 | 379.82 | 6.7 |
| SY-NP-1 | 5,523.75 | 36,824,970 | 5,506.73 | 96.65 | 99.69 | 339.00 | 6.1 |
| SY-NP-2 | 5,456.68 | 36,377,894 | 5,443.17 | 96.69 | 99.75 | 346.41 | 6.3 |
| SY-NP-3 | 5,337.42 | 35,582,780 | 5,322.30 | 96.53 | 99.72 | 324.48 | 6.1 |
| GZ-N-1 | 5,046.23 | 33,641,518 | 5,034.65 | 96.88 | 99.77 | 348.15 | 6.9 |
| GZ-N-2 | 5,359.48 | 35,729,872 | 5,341.24 | 96.77 | 99.66 | 365.11 | 6.8 |
| GZ-N-3 | 5,466.47 | 36,443,124 | 5,442.94 | 96.80 | 99.57 | 360.92 | 6.6 |
| GZ-PN-1 | 5,110.20 | 34,068,030 | 5,101.05 | 96.79 | 99.82 | 347.49 | 6.8 |
| GZ-PN-2 | 5,618.25 | 37,454,984 | 5,606.21 | 97.16 | 99.79 | 383.69 | 6.8 |
| GZ-PN-3 | 5,583.71 | 37,224,736 | 5,573.10 | 96.88 | 99.81 | 388.87 | 7.0 |
| GZ-P-1 | 5,612.96 | 37,419,742 | 5,600.71 | 94.93 | 99.78 | 192.60 | 3.4 |
| GZ-P-2 | 5,079.97 | 33,866,470 | 5,069.98 | 94.78 | 99.80 | 156.28 | 3.1 |
| GZ-P-3 | 5,354.43 | 35,696,188 | 5,342.30 | 94.87 | 99.77 | 162.80 | 3.0 |

**Table S3.** The KO number, function descriptions, gene name and classification of the investigated genes referring to KEGG database.

| Classification |  | KO number | Details for gene function | Corresponding gene |
| --- | --- | --- | --- | --- |
|  |  | K02039 | phoR/phoB inhibitor protein phoU | *phoU* |
| Genes involved in P-starvation response regulation |  | K07636 | two-component system, OmpR family, phosphate regulon sensor histidine kinase PhoR | *phoR* |
|  |  | K07657 | two-component system, OmpR family, phosphate regulon response regulator PhoB | *phoB* |
|  |  | K05813 | Glycerol-3-phosphate transporter subunit, periplasmic-binding component | *ugpB* |
|  |  | K05814 | Glycerol-3-phosphate transporter subunit | *ugpA* |
|  |  | K05815 | Glycerol-3-phosphate transporter subunit, membrane component | *ugpE* |
|  |  | K05816 | Glycerol-3-phosphate transporter subunit, ATP-binding component | *ugpC* |
| Genes involved in P-uptake and transport system |  | K02041 | phosphonate transport system, ATP-binding component | *phnC* |
|  |  | K02042 | phosphonate transport system, membrane component | *phnE* |
|  |  | K02044 | phosphonate transport system, periplasmic-binding component | *phnD* |
|  |  | K02036 | phosphate transport system, ATP-binding component | *pstB* |
|  |  | K02037 | phosphate transport system, membrane component | *pstC* |
|  |  | K02038 | phosphate transport system, membrane component | *pstA* |
|  |  | K02040 | phosphate transport system, periplasmic-binding component | *pstS* |
|  |  | K03306 | inorganic phosphate transporter | *pit* |
|  |  | K00117 | quinoprotein glucose dehydrogenase | *gcd* |
|  |  | K05774 | C-P lyase subunit, ribose 1,5-bisphosphokinase | *phnN* |
|  |  | K05780 | C-P lyase subunit, alpha-D-ribose 1-methylphosphonate 5-triphosphate synthase | *phnL* |
|  |  | K05781 | C-P lyase subunit, alpha-D-ribose 1-methylphosphonate 5-triphosphate synthase | *phnK* |
|  |  | K09994 | C-P lyase subunit, aminoalkylphosphonate N-acetyltransferase | *phnO* |
|  |  | K06162 | C-P lyase subunit, alpha-D-ribose 1-methylphosphonate 5-triphosphate diphosphatase | *phnM* |
|  |  | K06163 | C-P lyase subunit, alpha-D-ribose 1-methylphosphonate 5-phosphate C-P lyase | *phnJ* |
|  |  | K06164 | C-P lyase subunit, alpha-D-ribose 1-methylphosphonate 5-triphosphate synthase | *phnI* |
|  |  | K06165 | C-P lyase subunit, alpha-D-ribose 1-methylphosphonate 5-triphosphate synthase | *phnH* |
|  |  | K06166 | C-P lyase subunit, alpha-D-ribose 1-methylphosphonate 5-triphosphate synthase | *phnG* |
|  |  | K06167 | C-P lyase subunit, phosphoribosyl 1,2-cyclic phosphate phosphodiesterase | *phnP* |
| Genes involved in inorganic P-solubilization and organic P-mineralization |  | K02043 | C-P lyase subunit, GntR family transcriptional regulator, phosphonate transport system regulatory protein | *phnF* |
|  |  | K01524 | exopolyphosphatase / guanosine-5'-triphosphate,3'-diphosphate pyrophosphatase | *ppx* |
|  |  | K01507 | inorganic pyrophosphatase | *ppa* |
|  |  | K06193 | phosphonoacetate hydrolase | *phnA* |
|  |  | K05306 | phosphonoacetaldehyde hydrolase | *phnX* |
|  |  | K03430 | 2-aminoethylphosphonate-pyruvate transaminase | *phnW* |
|  |  | K01093 | 4-phytase | *appA* |
|  |  | K07048 | phosphotriesterase | *opd* |
|  |  | K01126 | glycerophosphoryl diester phosphodiesterase | *ugpQ* |
|  |  | K01077 | alkaline phosphatase | *phoA* |
|  |  | K01113 | alkaline phosphatase | *phoD* |
|  |  | K09474 | acid phosphatase (class A) | *phoN* |
|  |  | K03788 | acid phosphatase (class B) | *aphA* |
|  |  | K01078 | acid phosphatase | *olpA* |

**Table S4.** Results from two-way ANOVA with *p* values adjusted by Bonferroni-correction testing the effects of P input, sampling site and their interactions on the relative abundances of microbial P-transformation genes are presented. “*” and “n.s.” represent significant and non-significant differences at *p* < 0.05. The normality of residues and homogeneity of variance have been tested using Shapiro-wilk and levene test, respectively. Some observations that are not from normal distributions or do not have the same variance have been box-cox transferred prior to ANOVA.

| Gene name | Normality of residues (Shapiro-wilk) | Homogeneity of variance (levene test) | P input effects | Sampling site effects | P input × site effects |
| --- | --- | --- | --- | --- | --- |
| Genes involved in P-uptake and transport | Yes | Yes | n.s. | ***** | n.s. |
| Genes involved in inorganic P-solubilization and organic P-mineralization | Yes | Yes | n.s. | ***** | n.s. |
| *phoU* | Yes | Yes | n.s. | n.s. | n.s. |
| *phoR* | Yes | Yes | * | ***** | n.s. |
| *phoB* | Yes | Yes | n.s. | ***** | n.s. |
| *ugp* transporter systems | Yes | Yes | n.s. | ***** | n.s. |
| *phn* transporter systems | Yes | No | n.s. | ***** | n.s. |
| *pst* transporter systems | Yes | Yes | n.s. | ***** | n.s. |
| PQQGDH | Yes | Yes | n.s. | ***** | n.s. |
| C-P lyase subunit | Yes | Yes | n.s. | ***** | n.s. |
| Exopolyphosphatase | Yes | Yes | n.s. | n.s. | n.s. |
| Inorganic Pyrophosphatase | Yes | Yes | n.s. | n.s. | n.s. |
| Phosphonoacetate hydrolase | No | No | n.s. | ***** | n.s. |
| Phosphonoacetaldehyde hydrolase | No | Yes | n.s. | n.s. | n.s. |
| 2APT | No | No | n.s. | n.s. | n.s. |
| Phytase | No | No | n.s. | n.s. | n.s. |
| Phosphotriesterase | Yes | Yes | n.s. | n.s. | n.s. |
| GDP | Yes | Yes | n.s. | ***** | n.s. |
| Alkaline phosphatase | Yes | Yes | n.s. | ***** | n.s. |
| Acid phosphatase | Yes | Yes | n.s. | n.s. | n.s. |
| Inorganic phosphate transporter | Yes | Yes | ***** | ***** | n.s. |

**Table S5.** Results from two-way ANOVA with *p* values adjusted by Bonferroni-correction testing the effects of N input, sampling site and their interactions on the relative abundances of microbial P-transformation genes are presented. “*” and “n.s.” represent significant and non-significant differences at *p* < 0.05. The normality of residues and homogeneity of variance have been tested using Shapiro-wilk and levene test, respectively. Some observations that are not from normal distributions or do not have the same variance have been box-cox transferred prior to ANOVA.

| Gene name | Normality of residues (Shapiro-wilk) | Homogeneity of variance (levene test) | N input effects | Sampling site effects | N input × site effects |
| --- | --- | --- | --- | --- | --- |
| Genes involved in P-uptake and transport | Yes | Yes | ***** | ***** | ***** |
| Genes involved in inorganic P-solubilization and organic P-mineralization | Yes | Yes | ***** | ***** | n.s. |
| *phoU* | Yes | Yes | n.s. | n.s. | n.s. |
| *phoR* | Yes | Yes | ***** | ***** | ***** |
| *phoB* | Yes | Yes | n.s. | ***** | ***** |
| *ugp* transporter systems | Yes | Yes | n.s. | ***** | n.s. |
| *phn* transporter systems | Yes | No | ***** | ***** | n.s. |
| *pst* transporter systems | Yes | Yes | n.s. | ***** | n.s. |
| PQQGDH | Yes | Yes | ***** | ***** | n.s. |
| C-P lyase subunit | Yes | Yes | n.s. | n.s. | * |
| Exopolyphosphatase | Yes | Yes | n.s. | n.s. | n.s. |
| Inorganic Pyrophosphatase | Yes | Yes | n.s. | n.s. | n.s. |
| Phosphonoacetate hydrolase | No | No | n.s. | ***** | n.s. |
| Phosphonoacetaldehyde hydrolase | No | No | n.s. | n.s. | n.s. |
| 2APT | No | Yes | n.s. | ***** | n.s. |
| Phytase | Yes | Yes | ***** | n.s. | n.s. |
| Phosphotriesterase | Yes | Yes | n.s. | n.s. | ***** |
| GDP | Yes | Yes | n.s. | ***** | n.s. |
| Alkaline phosphatase | Yes | Yes | ***** | ***** | ***** |
| Acid phosphatase | Yes | Yes | n.s. | n.s. | n.s. |
| Inorganic phosphate transporter | Yes | Yes | n.s. | ***** | n.s. |

**Table S6.** Basic topological properties of gene occurrence networks with the treatments of -N, +N, -P and +P.

| Topological properties | Explanation | -P | +P | -N | +N |
| --- | --- | --- | --- | --- | --- |
| Average degree | The average number of edges connected to nodes in a network | 5.84 | 5.52 | 7.67 | 4.545 |
| Graph density | The ratio of the number of edges and the maximal number of edges in a network | 0.243 | 0.23 | 0.333 | 0.216 |
| Modularity | The strength of the division of a network into functionally independent modules | 0.197 | 0.353 | 0.106 | 0.217 |
| Average clustering  coefficient | The average of the probabilities that the adjacent nodes of a node are connected | 0.665 | 0.576 | 0.843 | 0.717 |
| Average path length | The average number of steps to get from one node of the network to another. | 2.303 | 2.527 | 1.506 | 1.718 |

**Table S7.** Coefficients (*r*) of Spearman's correlations between the relative abundance of genes responsible for *ugp*, *phn*, *pst* and *gcd* and soil N:P and P forms across four experimental sites. **, *p* <0.01; *, *p* <0.05. Al-P, Phosphorus bound to aluminum; Fe-P, Phosphorus bound to iron; O-P, Occluded phosphate; Ca-P, Calcium phosphates.

.

| Genes | N:P | Al-P | Fe-P | O-P | Ca-P |
| --- | --- | --- | --- | --- | --- |
| *ugpABCE* | -0.57** | 0.57** | 0.58** | 0.68** | 0.61** |
| *phnCDE* | -0.34** | 0.27 | 0.55** | 0.44** | 0.42* |
| *pstABCS* | -0.55** | 0.50** | 0.53** | 0.53** | 0.46** |
| *gcd* | -0.42* | 0.53** | 0.49** | 0.52** | 0.45** |


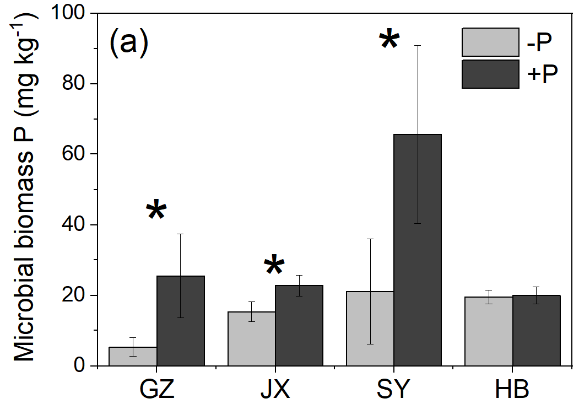

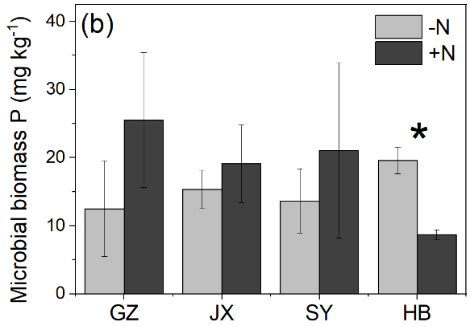


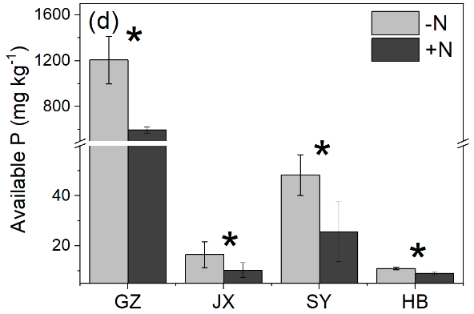

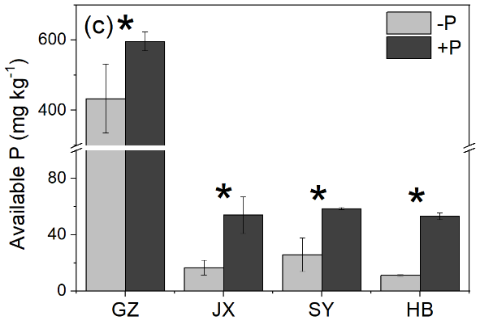


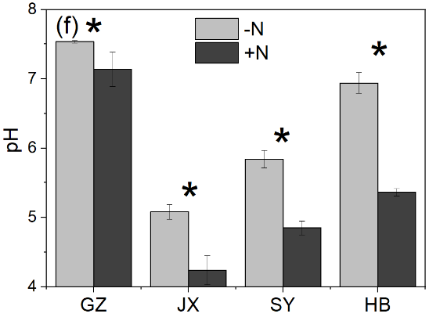

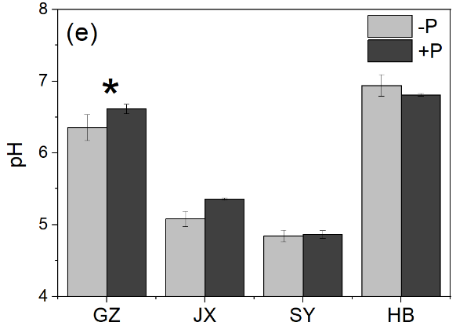


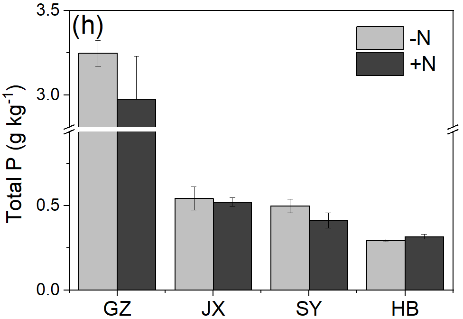

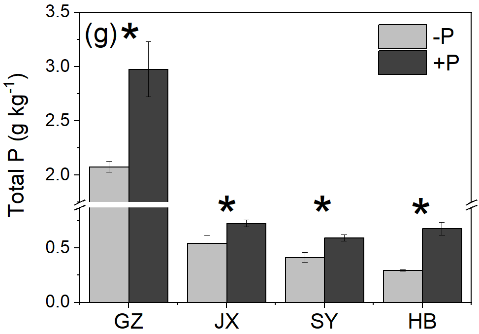


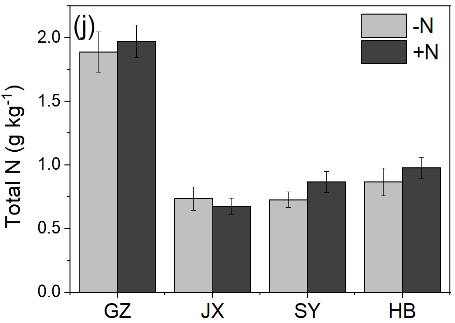

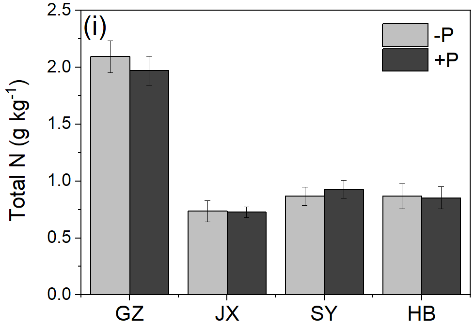


**Fig. S1.** Effects of long-term N and P input on soil microbial biomass P, P availability, pH, total P and total N at the four experimental sites. Significant differences in soil parameters at individual sites by one-way ANOVA are shown with “*” at *p* < 0.05. Error bars are ± standard error.


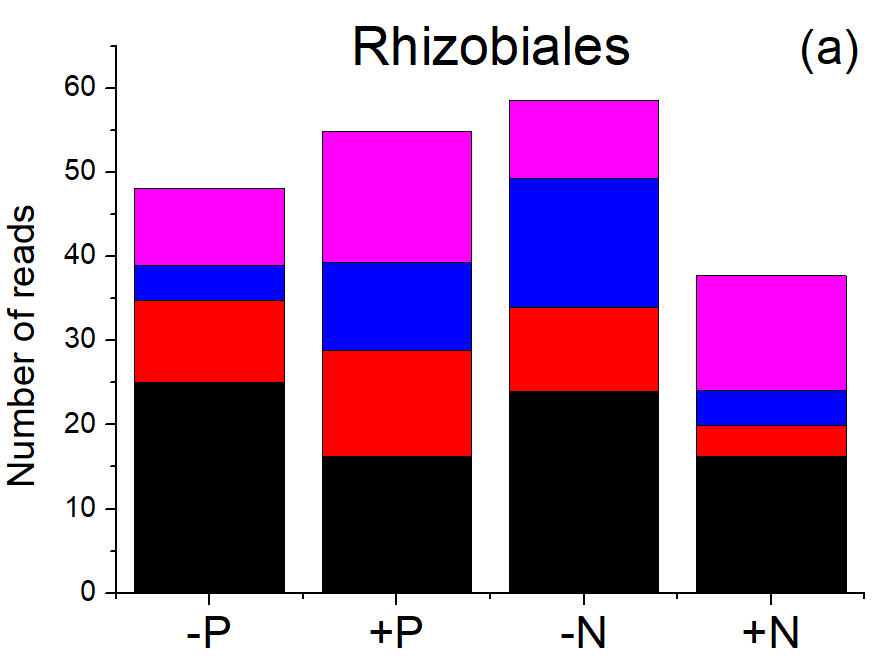


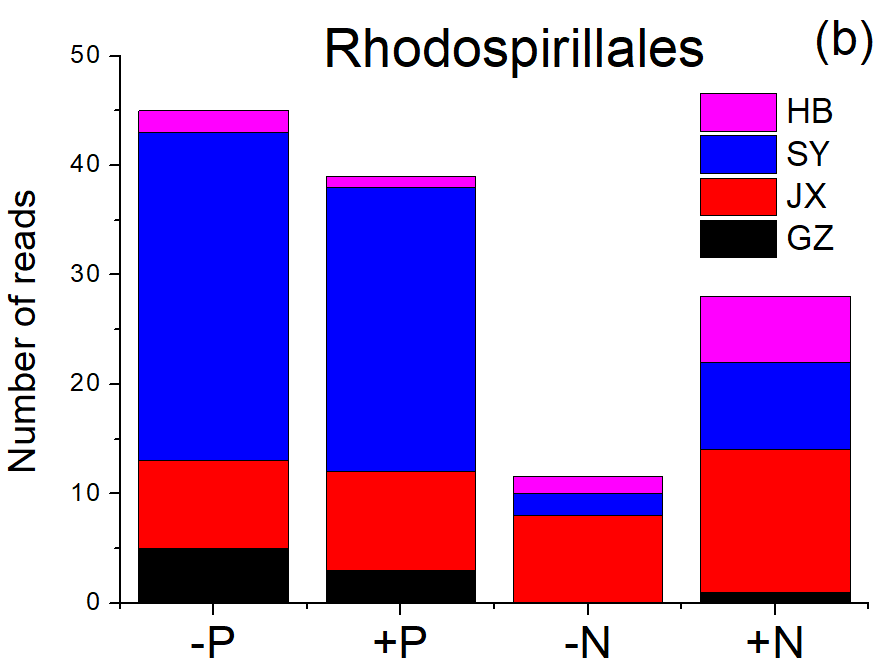


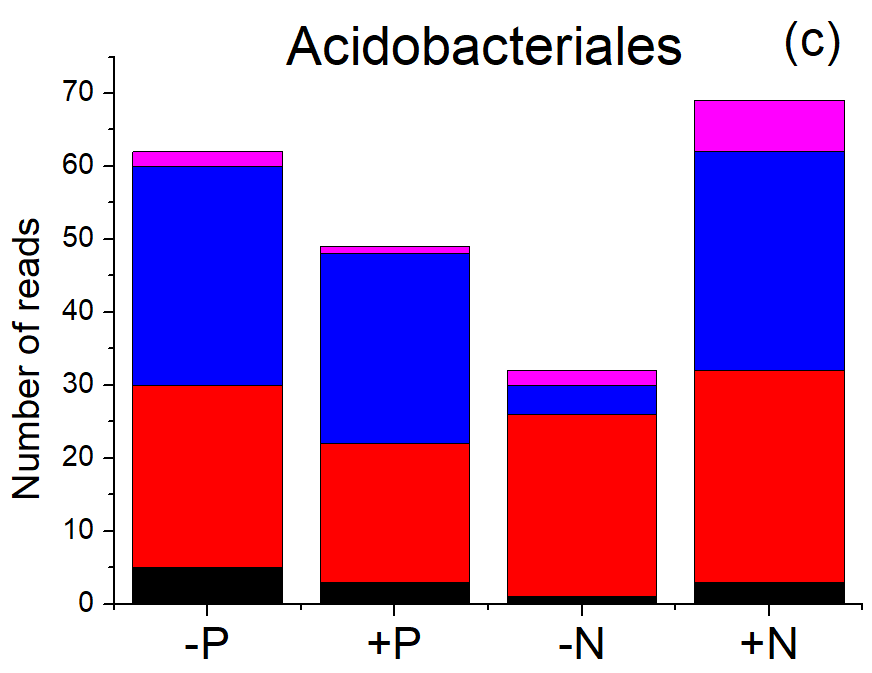


**Fig. S2.** Taxonomic assignments of genes involved in P-solubilization and mineralization at the order level at each experimental site with and without N or P input. (a) *Rhizobiales*, (b) *Rhodospirillales* and (c) *Acidobacteriales*. These microorganisms were presented based on the criterion that at least three of the four sites showed consistent trends in abundance change.


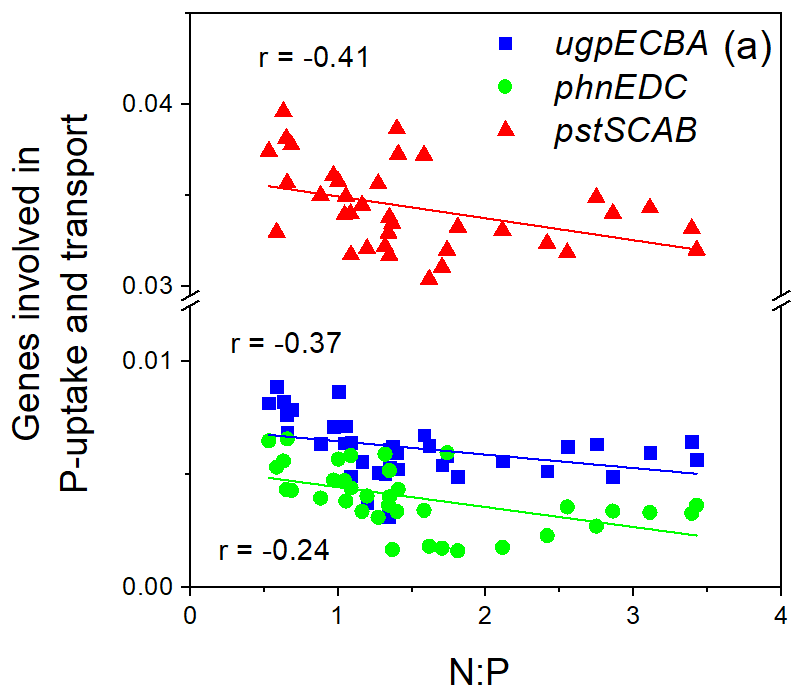

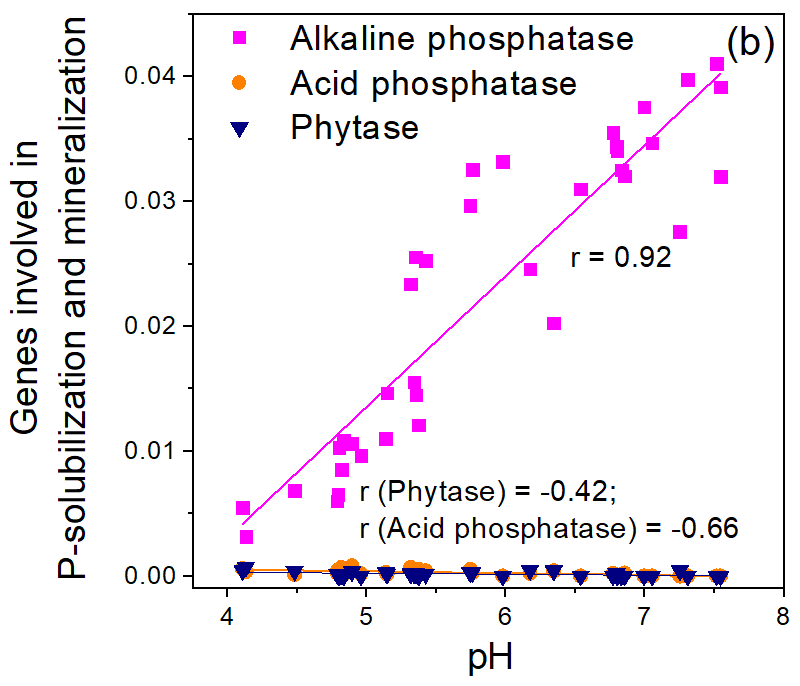


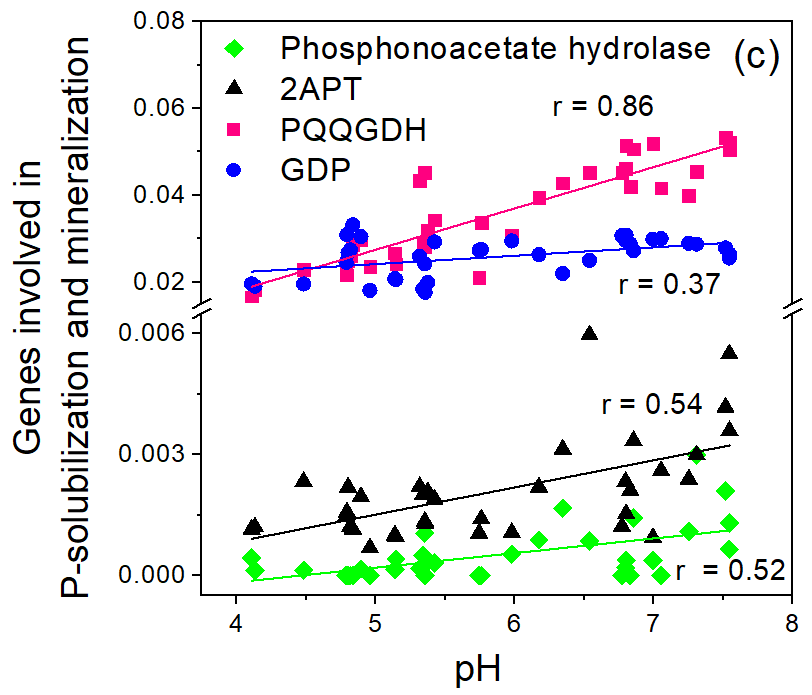

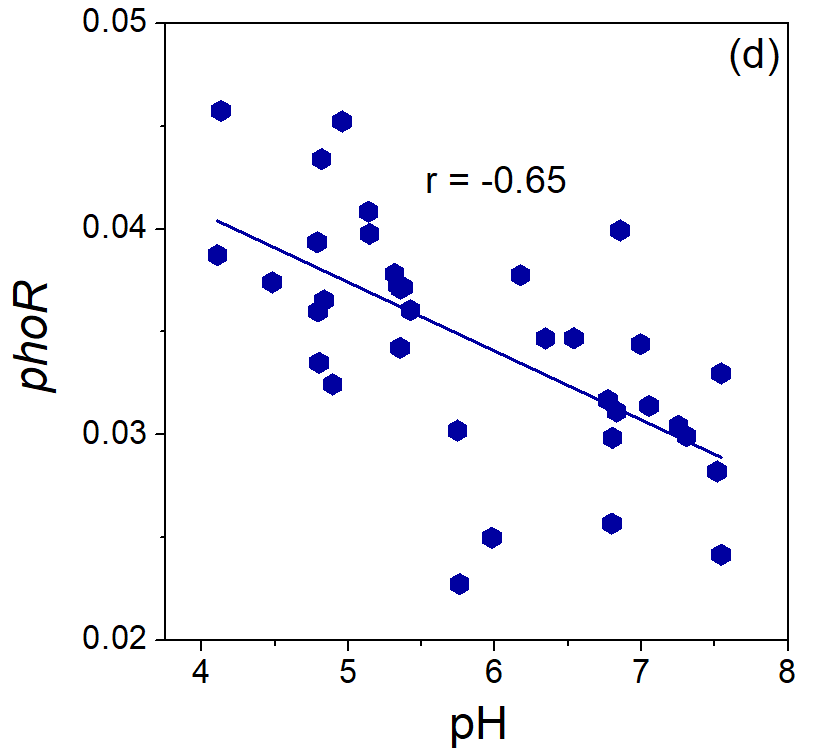


**Fig. S3.** Spearman's correlations between the relative abundance of the genes involved in P-uptake and transport and soil N:P ratios (a), between genes involved in P-solubilization and mineralization and soil pH (b & c), and between P-starvation response regulation (*phoR*) and soil pH (d).

No significant correlations between genes involved in P-solubilization and mineralization and N:P, or between genes involved in P-uptake and transport and pH were observed.

2APT, 2-aminoethylphosphonate-pyruvate transaminase: *phnW*; GDP, glycerophosphoryl diester phosphodiesterase: *ugpQ*; PQQGDH, quinoprotein glucose dehydrogenase: *gcd*; Alkaline phosphatase: *phoD* and *phoA*; Acid phosphatase: *phoN*, *aphA* and *olpA*; Phytase: *appA*; phosphonoacetate hydrolase: *phnA*.
